# Photoelectrochemical Enhancement of Graphene@WS_2_ Nanosheets for Water Splitting Reaction

**DOI:** 10.3390/nano12111914

**Published:** 2022-06-03

**Authors:** Mahmoud Nasr, Lamyae Benhamou, Ahmed Kotbi, Nitul S. Rajput, Andrea Campos, Abdel-Ilah Lahmar, Khalid Hoummada, Khaled Kaja, Mimoun El Marssi, Mustapha Jouiad

**Affiliations:** 1 Laboratory of Physics of Condensed Matter, University of Picardie Jules Verne, Scientific Pole, 33 Rue Saint-Leu, CEDEX 1, 80039 Amiens, France; mnldmohammed@gmail.com (M.N.); lamyae.benhamou@etud.u-picardie.fr (L.B.); ahmed.kotbi@u-picardie.fr (A.K.); abdel.ilah.lahmar@u-picardie.fr (A.-I.L.); mimoun.elmarssi@u-picardie.fr (M.E.M.); 2 Solid-State Physics Department, Physics Research Institute, National Research Centre, 33 El-Bohouth Street, Dokki, Giza 12622, Egypt; 3 Advanced Materials Research Center, Technology Innovation Institute, Abu Dhabi P.O. Box 9639, United Arab Emirates; nitul.rajput@tii.ae; 4 Aix Marseille University, Faculté des Sciences et Techniques, CP2M, IM2NP, Avenue Escadrille Normandie Niemen, 13397 Marseille, France; andrea.campos@univ-amu.fr (A.C.); khalid.hoummada@univ-amu.fr (K.H.); 5 Laboratoire National de Métrologie et D’essais (LNE), 29 Avenue Roger Hannequin, 78197 Trappes, France; khaled.kaja@lne.fr

**Keywords:** WS_2_ nanosheets, graphene@WS_2_, photochemical measurements, water splitting, reversible hydrogen electrode (RHE), photoanode

## Abstract

Tungsten disulfide nanosheets were successfully prepared by one-step chemical vapor deposition using tungsten oxide and thiourea in an inert gas environment. The size of the obtained nanosheets was subsequently reduced down to below 20 nm in width and 150 nm in length using high-energy ball milling, followed by 0.5 and 1 wt% graphene loading. The corresponding vibrational and structural characterizations are consistent with the fabrication of a pure WS_2_ structure for neat sampling and the presence of the graphene characteristic vibration modes in graphene@WS_2_ compounds. Additional morphological and crystal structures were examined and confirmed by high-resolution electron microscopy. Subsequently, the investigations of the optical properties evidenced the high optical absorption (98%) and lower band gap (1.75 eV) for the graphene@WS_2_ compared to the other samples, with good band-edge alignment to water-splitting reaction. In addition, the photoelectrochemical measurements revealed that the graphene@WS_2_ (1 wt%) exhibits an excellent photocurrent density (95 μA/cm^2^ at 1.23 V bias) compared with RHE and higher applied bias potential efficiency under standard simulated solar illumination AM1.5G. Precisely, graphene@WS_2_ (1 wt%) exhibits 3.3 times higher performance compared to pristine WS_2_ and higher charge transfer ability, as measured by electrical impedance spectroscopy, suggesting its potential use as an efficient photoanode for hydrogen evolution reaction.

## 1. Introduction

Hydrogen fuel is considered a promising source of clean energy that could partly replace fossil fuels at the origin of greenhouse gas emissions. Today, there is a growing universal tendency to focus research and development on green hydrogen production by considering the general ecosystem to lower its costs and make it a more affordable clean-energy source, especially through water splitting (WS) [1,2,3,4,5]. In this context, several advanced materials were successfully examined and tested for enhancing the WS reaction, such as noble metals combined with highly catalytic semiconductors, which have shown an improved hydrogen evolution reaction (HER). However, their complex implementation and high cost prohibited their adoption in realistic WS plants [6,7,8,9]. Therefore, cost-efficient and easy-to-produce alternative materials with improved WS performances have attracted a great interest. To this end, WS_2_, a transition-metal dichalcogenide (TMD) semiconductor, constitutes a highly attractive option owing to its excellent band gap tunability, its stability at high temperatures in corrosive media, and its easy and low-cost fabrication. Recently, both computational and experimental studies carried out on WS_2_ have shown its effective role in catalyzing HER with a relatively good yield [10,11,12,13,14,15,16]. WS_2_-like materials, such as vertically aligned MoS_2_, have shown increasingly exposed edges as active sites for HER electrocatalysis, while the basal plane is catalytically inert [17]. The increasing interest in using TMDs as supporting electrodes is justified by their easy and low-cost process [15,18,19,20]. To further improve WS_2_ performances, it is crucial to select an appropriate catalyst-supporting material that can prevent high-surface-area WS_2_ nanostructures from agglomerating, enhance electron mobility, and overcome the surface overpotential to improve the overall HER performance. Graphene is the other two-dimensional carbon material that would meet the aforementioned criteria. Due to its high ability in electronic excitation and mobility, its mechanical properties, and its high stability, even at the nanoscale, graphene has received rigorous scientific and industrial attention for a long time [21,22,23,24,25,26]. Indeed, graphene is reported to possess high specific surface area (~2600 m^2^/g), high electron mobility μe at room temperature (250,000 cm^2^/Vs), outstanding thermal conductivity (5000 W m^−1^ K^−1^), and high electrical conductivity (5:6.4 × 10^6^ S/m), as well as a very low weight. These outstanding proprieties enable graphene to be considered an excellent electrocatalyst support for WS_2_ nanostructures [21,22].

In this work, we first produced low-cost graphene@WS_2_ nanocomposite as electrodes for improved WS reactions. Next, we conducted a systematic structural characterization and assessment of the optical properties to evaluate the performance of the fabricated samples in photocatalysis-driven WS.

## 2. Materials and Methods

The raw materials, namely tungsten oxide (WO_3_) and thiourea (CH_4_N_2_S) powders, were acquired from Alfa Aesar™ (Thermo Fisher GmbH, Kandel, Germany). Graphene nanoplatelets were purchased from Sigma Aldrich™ St. Louis, MO, USA. The indium tin oxide (ITO) substrates used in this study are from Lumtec™ (Tapei, Taiwan), exhibiting a resistance < 10 Ω/cm^2^. All used substrates were cleaned by immersion in detergent, rinsed successively with acetone, ethanol, and deionized water, and dried under nitrogen flow. WS_2_ nanosheets were prepared using four processing conditions consisting of various WO_3_ and thiourea contents until achieving the complete suppression of the oxygen residue and obtaining pure WS_2_ nanosheets. The initial materials’ contents and the corresponding samples are summarized in the Table 1.

**Figure 1:**
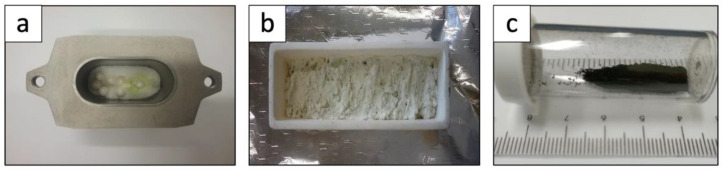
(**a**) Tungsten carbide container including sample mixture and ZrO_2_ balls; (**b**) ball-milled mixture collected in alumina boat; (**c**) WS_2_ nanosheets obtained after CVD reaction.

In a typical processing route, WS_2_ nanosheets are prepared by mixing WO_3_ with thiourea using a high-energy ball milling process E-Max Retsh™ (GmbH, Düsseldorf, Germany) machine with tungsten carbide container and Zirconia oxide balls (Figure 1a). After milling at 400 rpm for 30 min, the obtained mixture was collected into 50 mL alumina crucible (Figure 1b). A tube furnace was used for the chemical vapor deposition (CVD) reaction. The processing temperature was set to 850 °C under nitrogen gas flow of 500 sccm at 20 °C/s heating rate. The mixture was introduced into the tube furnace at T = 400 °C, and the CVD reaction took place for 1 h of dwell time at 850 °C, followed by air cooling. The obtained WS_2_ exhibited its typical black color (Figure 1c).

The chemical reaction occurring during the CVD process is expressed as follows:

$${\mathrm{WO}}_{3\;}+2\left({\mathrm{CH}}_{4}N_{2}S\right)\to \frac{\Delta }{N_{2}}\to {\;\mathrm{WS}}_{2\;}+2\left({\mathrm{CH}}_{4}N_{2}\right)+{\;O}_{3},$$

**Figure 2:**
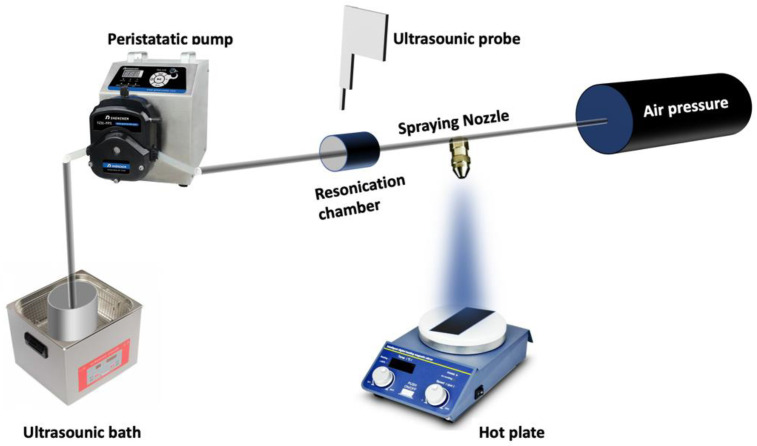
Schematic diagram of the spray pyrolysis system used to prepare thin films out of the graphene@WS_2_.

For the graphene@WS_2_ nanocomposites, two graphene contents (0.5 wt% and 1 wt%) were added to the optimized fabricated WS_2_ nanosheets (S4), followed by high-energy ball milling at 400 rpm for 1 h. To investigate the different physical properties of the resulting compounds, thin films were prepared on top of various substrates, such as glass, quartz, and ITO. Figure 2 shows an illustration of the enhanced spray technique, where two ultrasonic-solution dispersive devices were utilized to prevent WS_2_ nanosheets from aggregating in the transferring tube. Ethanol was used as the host solution.

For all samples, the spraying procedure consisted of 0.1 g of a mixture made of WS_2_ with or without graphene, which was poured into a 40-megaliter ethanol container followed by water-bath sonication, as shown in Figure 2. During the solution distribution through a peristatic pump, a second sonication (ultrasound probe) was used to resuspend the mixture towards the nozzle. Finally, an air pressure of 1.2 bar allowed spraying the dispersed suspension though a nozzle over the desired substrate, which was kept at 45 °C with a hot plate. The nozzle–substrate distance was maintained at 20 cm and the deposition rate was set to 1 mL/min for a total deposition time of 30 min. The obtained film thickness was in the range of 150 to 200 nm.

Subsequently, the structural characterization was carried out using a Bruker™ D4 Endeavor X-ray diffractometer with a 1.54 Å CuKα source, and the vibrational analyses were performed with Raman spectroscopy Renishaw™ ( Wotton-under-Edge, UK) using a green laser excitation source (532 nm). The microstructure analysis was performed with a Zeiss™ (Oberkochen, Germany) Gemini 500 ultra-high resolution field emission electron microscope (FESEM) operating at low voltage (1 kV), using an In-lens detector. The crystal structure and the morphology of the obtained nanocomposite were investigated by high-resolution transmission electron microscopy (HRTEM) Cs-corrected Titan from Thermo Fisher Scientific™ (Waltham, MA, USA) operating at 300 kV. TEM samples were prepared on holey carbon Cu grids using drop-casting method. The analysis of optical properties was conducted on a UV-vis-near IR spectrometer V-700 JASCO™ (Easton, MD, USA) and Fourier transform infrared spectroscopy (FTIR) from Thermo Fisher Scientific™ (Waltham, MA, USA). The electrical impedance spectroscopy (EIS) and the photoelectrochemical (PEC) measurements were performed using PalmSens4™ (Houten, Netherlands) EIS electrochemical interface.

## 3. Results and Discussion

### 3.1. XRD Analysis

**Figure 3:**
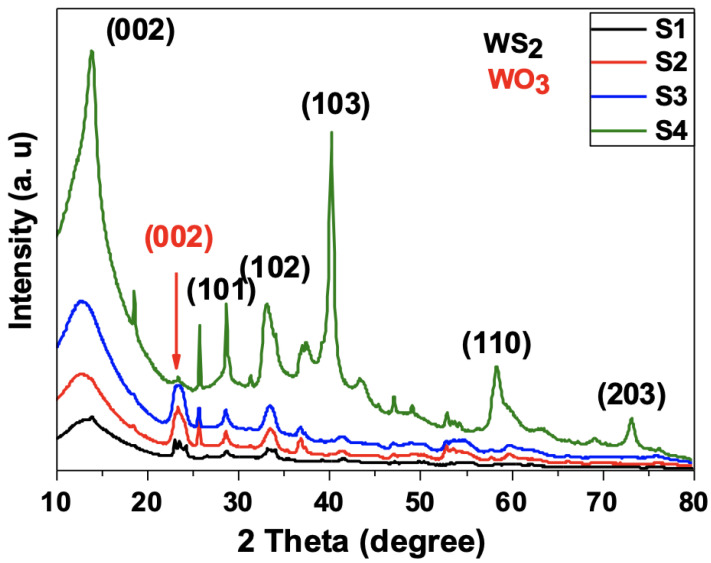
XRD diagrams for the four processed samples. Note the absence of WO_3_ peak in sample S4 indicating the high purity WS_2_ nanosheets.

The XRD diagrams obtained for the four prepared WS_2_ samples are shown in Figure 3. The XRD patterns were recorded in the range of 2-theta 10–80° and compared to the observed reflection planes (002), (101), (102), (103), (110) and (203) with the standard diffraction data file (JCPDF card no. 01-084-1398). These diffraction peaks were indexed with the hexagonal WS_2_ phase of cell constants a = b = 3.15 Å, c = 12.32 Å.

The XRD diagrams of the samples S1, S2, and S3 exhibit an extra peak at (002) position which corresponds to the WO_3_ structure. This indicates that the reaction of the WO_3_ with the thiourea was not complete; hence, the resulting WS_2_ of these samples contained WO_3_ residue. It is worth noting that the strength of the WO_3_ residue peak was observed to decrease with increasing thiourea content (from sample S1 to S3). By contrast, the WO_3_ peak was no longer visible at higher thiourea content as it disappeared for the sample S4. This result suggests that the excess thiourea added to the mixture made it possible to obtain the pure WS_2_ nanosheets.

### 3.2. Raman Spectroscopy

**Figure 4:**
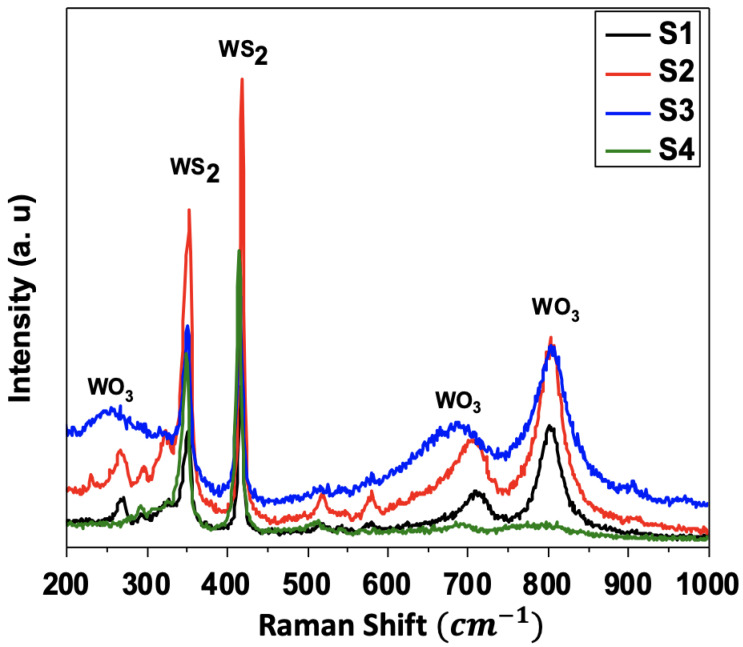
Raman spectra for the four prepared WS_2_ samples. Note the absence of WO_3_ vibration modes at 286 cm^−1^, 666 cm^−1^, and 820 cm^−1^ in sample S4, which highlights the high purity of WS_2_.

Figure 4 illustrates the vibrational modes of the four processed WS_2_ samples. All the samples show common WS_2_ characteristic peaks, i.e., two strong peaks attributed to E_2g1_ in-plane and A_1g_ out-of-plane vibrational modes appearing at the 350 and 420 cm^−1^ positions, respectively. The E_2g1_ mode involves a displacement of W and S atoms, whereas the A_1g_ mode concerns only the S atoms.

Similar to the XRD results, the Raman spectra of the S1, S2, and S3 showed the presence of WO_3_ vibration modes at the 293 cm^−1^, 675 cm^−1^, and 836 cm^−1^ positions, respectively, while the S4 only exhibited the vibration modes E^1^_2g_ and A_1g_ for WS_2_ occurring at 351 cm^−1^ and 415 cm^−1^, respectively. As mentioned above, these results confirm that the CVD reaction was fully completed for the S4 and the oxygen was entirely consumed.

**Figure 5:**
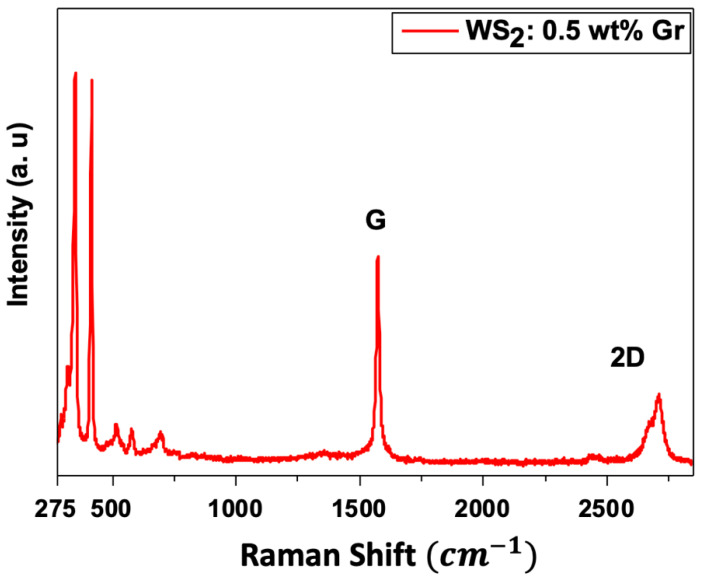
Raman spectra for graphene@WS_2_ samples. Typical G-band and 2D-band vibrational modes of graphene are recorded.

Furthermore, the Raman spectroscopy was carried out on graphene@WS_2_ samples, and the typical Raman spectra are depicted in Figure 5. In addition to the presence of the common WS_2_ peaks, graphene vibrational modes were also obtained for both samples at 1560–1575 cm^−1^ and at 2647–2700 cm^−1^ for the G-band and 2D-band, respectively. This confirms the presence of the graphene in the processed graphene@WS_2_ samples.

### 3.3. FTIR Spectroscopy

**Figure 6:**
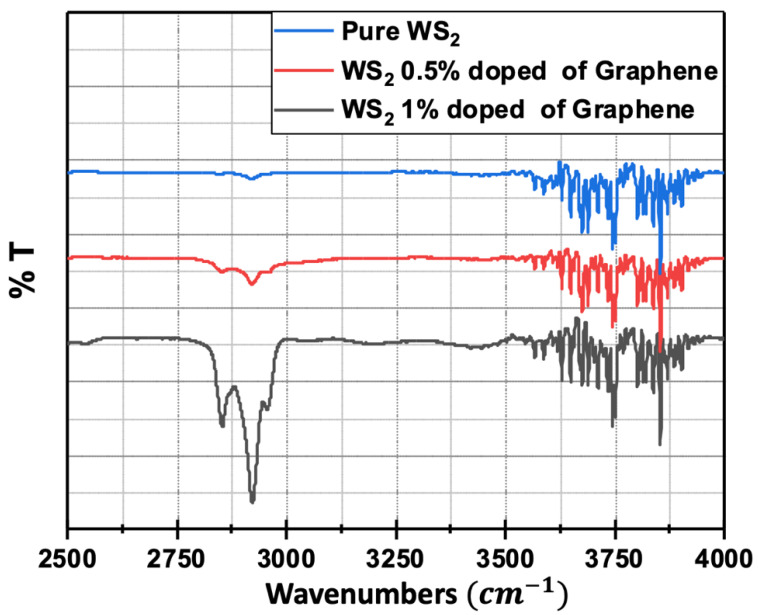
FTIR absorption spectra of WS_2_, WS_2_: 0.5 wt% Gr, and WS_2_: 1 wt% Gr. Note the presence of the typical graphene absorption peak at 2780–3000 cm^−1^ for both graphene@WS_2_ samples.

To further screen the presence of the graphene in the processed graphene@WS_2_ nanocomposite samples, FTIR absorption spectroscopy was conducted on pure WS_2_, WS_2_: 0.5 wt% Gr, and WS_2_: 1 wt% Gr, respectively, as shown in Figure 6.

As can be seen, the typical graphene absorption peak is present in the graphene@WS_2_ nanocomposites and the strength of this peak is more pronounced for the sample with the higher graphene content.

### 3.4. Microstructure Analysis

**Figure 7:**
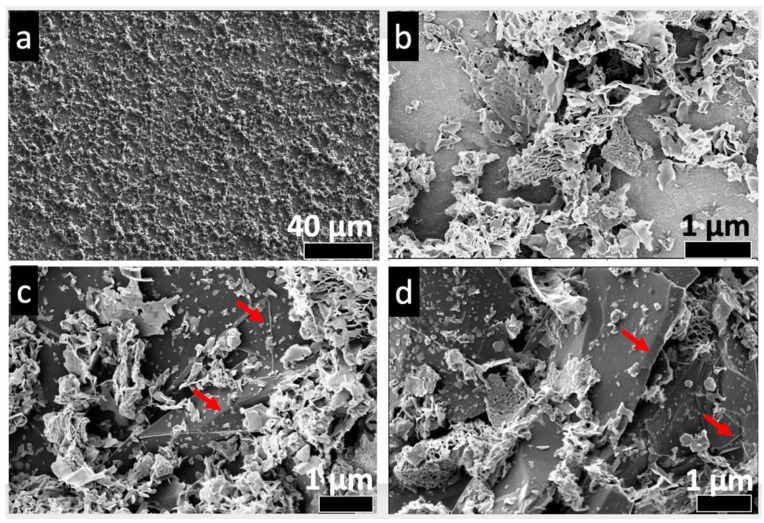
Low kV SEM micrographs: (**a**) typical general view for all samples; (**b**) zoom-in on neat WS_2_ nanosheets; (**c**) zoom-in on WS_2_: 0.5 wt% sample; and (**d**) zoom-in on WS_2_: 1 wt% sample. Red arrows identify the graphene platelets on grapahene@WS_2_ samples.

A general view of the sprayed samples on the ITO substrate is given in Figure 7a. As can be seen, the nanosheets are evenly distributed with relatively uniform thickness. The insets shown in Figure 7b–d highlight the inner structures of the neat WS_2_, WS_2_: 0.5 wt% Gr, and WS_2_: 1 wt% Gr, respectively. Both the WS_2_ nanosheets and the graphene platelets are visible. These samples were further investigated by HRTEM and used for the photoelectrochemical measurements.

**Figure 8:**
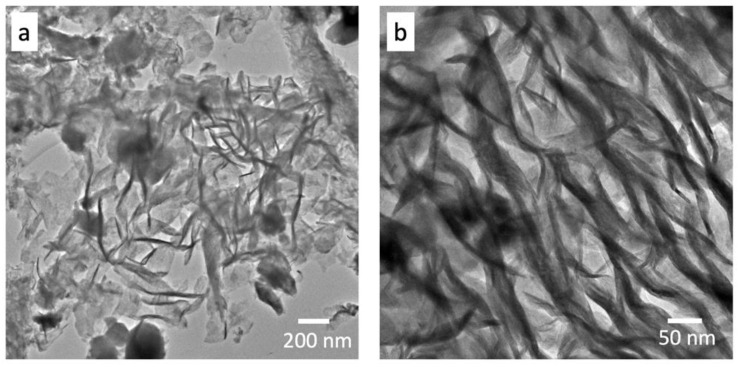
Bright-field TEM images of graphene@WS_2_ nanocomposites: (**a**) low and (**b**) high magnification.

Figure 8 depicts the bright-field TEM image of the typical microstructure encountered in WS_2_: 1 wt% Gr. The WS_2_ nanosheets (dark contrast) appeared to be encapsulated in the graphene nanoplatelets (light contrast), as shown in Figure 8a. A higher-magnification TEM image is given in Figure 8b; it shows a focus on entangled graphene@WS_2_ nanosheets. The quality of the nanocomposite was further verified using HRTEM.

**Figure 9:**
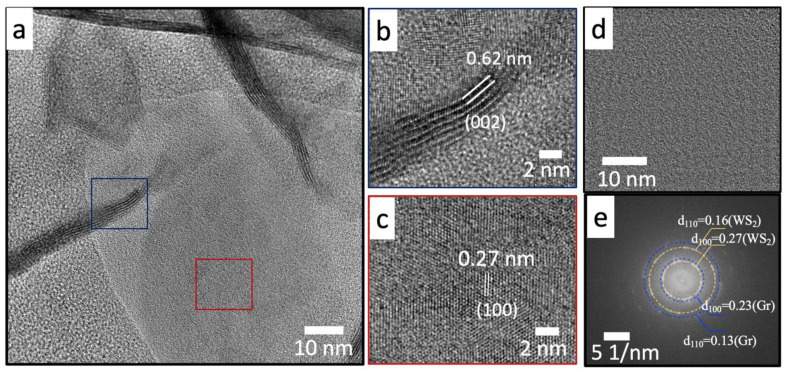
HRTEM images of graphene@WS_2_ nanocomposites: (**a**) general view; (**b**) blue-box zoom-in, showing the crystal lattice of WS_2_ d_002_; (**c**) red-box zoom-in showing the crystal lattice of WS_2_ d_100_; (**d**) HRTEM image of interconnected graphene ws2 region and corresponding (**e**) FFT image.

Figure 9a clearly shows the interconnections between the WS_2_ nanosheets and the graphene nanoplatelets, delimited by the blue box highlighted in Figure 9b. Their corresponding crystal structure conformed with the 2H-WS_2_ materials, as demonstrated by the interplanar distances d_(002)_ = 0.62 nm and d_(100)_ = 0.27 nm, illustrated in the red box in Figure 9c. The analysis of the interconnected WS_2_ and graphene regions given in Figure 9d revealed the presence of both graphene and WS_2_, as demonstrated by the corresponding fast Fourier transform (FFT) intensity image depicted in Figure 9e.

### 3.5. Optical Properties

**Figure 10:**
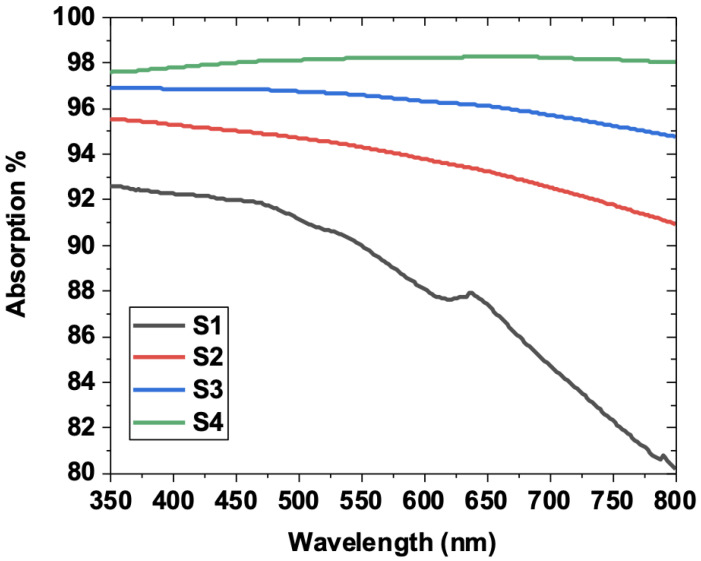
Optical absorption for the four considered samples. Note excitons position at 620 nm.

The investigation of the optical properties was carried out using an optical spectrometer in the 350–800 nm range. Figure 10 shows the optical absorption of all the considered samples.

**Figure 11:**
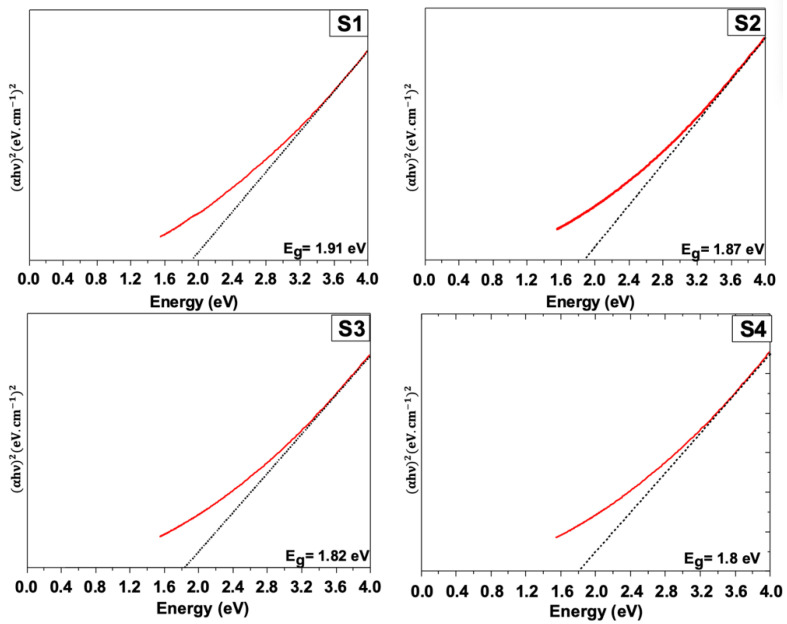
Tauc plots for all samples showing the lowest band-gap energy value for the sample S4.

As can be seen, samples S1, S2, and S3 exhibited similar optical behavior, consisting of a steady decrease in the optical absorption, which started at 93–97% and hit 80% for S1. This suggests that the presence of WO_3_ impurities induces a decrease in the optical absorption in the visible region. By contrast, the optimized sample S4 showed a relatively unchanged optical absorption. In particular, it remained stable until it reached the excitons position (around 620 nm), at which point it slightly increased. It is clear that the optimized WS_2_ sample S4 exhibited high broadband light absorption in the visible region, reaching more than 98% compared to the other samples. Using the Tauc formula (αhυ)^n^ = A(hϑ − Eg), the band-gap energy was obtained, as depicted in Figure 11.

The obtained band-gap energies were 1.91 eV, 1.87 eV, 1.82 eV, and 1.8 eV for S1, S2, S3, and S4, respectively. The S4 showed the lowest energy, which was in agreement with the broadband optical absorption performances achieved.

**Figure 12:**
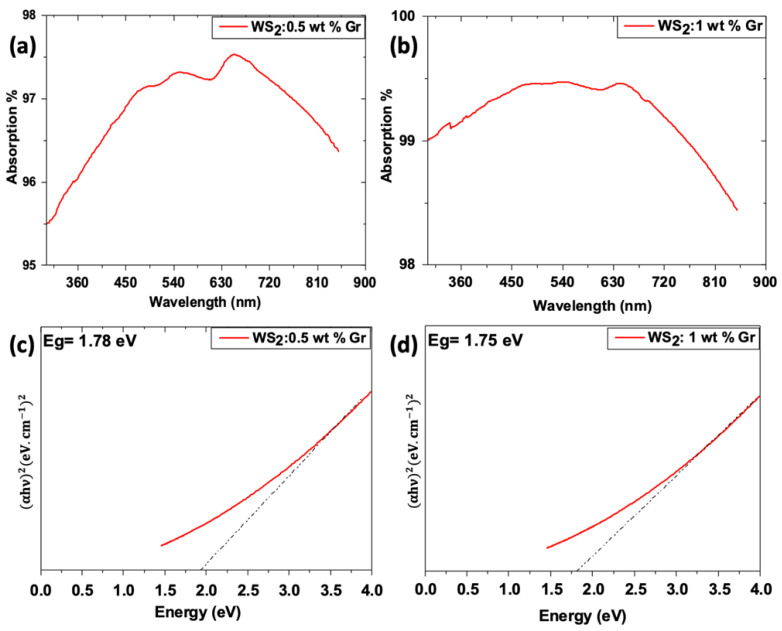
Optical properties of graphene@WS_2_ samples: (**a**,**b**) optical absorption of WS_2_ 0.5 wt% and 1 wt%, respectively; (**c**,**d**) corresponding Tauc plots.

To evaluate the effect of the graphene’s incorporation on the WS_2_ nanosheets, the optical absorption was also measured for both graphene@WS_2_ samples, namely 0.5 wt% Gr and 1 wt% Gr, and the results are shown in Figure 12.

The addition of WS_2_: 0.5 wt% Gr does not seem to enhance the optical absorption (Figure 10 vs. Figure 12a). Indeed, the optical absorption of this sample reaches 98% absorption but only in the 550–750 nm region in contrast to pristine WS_2_, which exhibits a broadband absorption. Nevertheless, the WS_2_: 0.5 wt% Gr band gap energy appears to slight decrease to 1.78 eV (Figure 12c). In contrast, the WS_2_: 1 wt% Gr sample does exhibit an optical absorption exceeding 99% across the entire visible light spectrum. Moreover, its band gap energy is further decreased to reach 1.75 eV. Considering these performances, WS_2_ and WS_2_: 1 wt% Gr samples were selected to perform photochemical measurements.

### 3.6. Photochemical Measurements

In this section, the photocatalytic performances of neat WS_2_ and WS_2_: 1 wt% Gr samples are screened. Both samples were deposited on ITO substrate and immersed in deionized water medium (pH = 6). Then, a linear sweep voltammetry (LSV) and chronoamperometry experiments were carried under standard solar simulator (energy~AM1.5G).

To extract the potential of RHE, the following Nernst equation was used:

$$E_{\mathrm{RHE}}=E_{\left(\mathrm{Ag}/\mathrm{AgCl}\right)}+\left(0.059\times \;\mathrm{pH}\right)+E_{0}$$

where *E*_0_ ≈ 0.197 V at 25 °C and *E*_Ag/AgCl_ is the applied potential.

**Figure 13:**
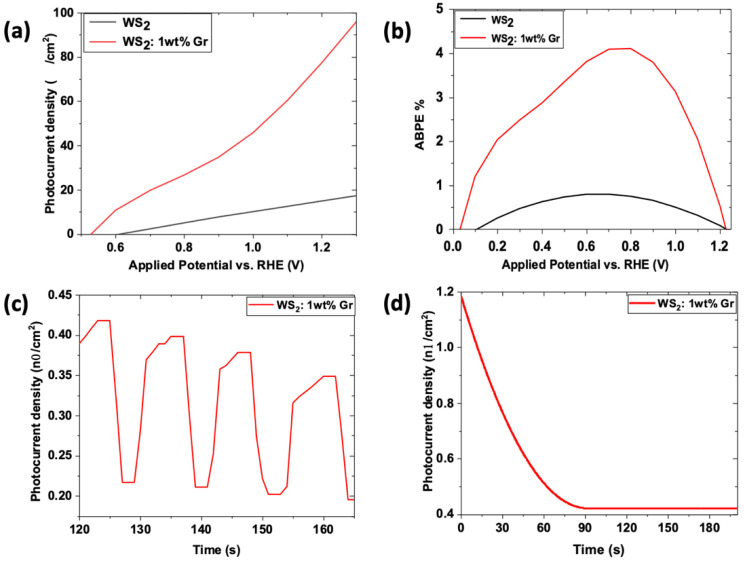
Photoelectrochemical measurements carried out on optimized WS_2_ S4 and graphene@WS_2_: 1 wt%: (**a**) generated current density as function of applied potential vs. RHE; (**b**) applied bias potential efficiency as function of applied bias with respect to RHE. Chronoamperometry experiment performed on WS_2_: 1 wt% Gr using solar simulator of AM1.5G: (**c**) cyclic and (**d**) steady state tests.

For both samples, the active surface is about 1 cm^2^ and the distance between the three electrodes was kept at 5 mm. A Pt fishnet (0.5 cm outer diameter) was used as counter electrode, while standard Ag/AgCl was used as the reference electrode. The LSV scan rate was set to 0.1 V/s along 0–1.5 V range. Prior to LSV experiments, all measurements were stabilized for 200 s under zero applied potential. The results are depicted in Figure 13.

As can be seen in Figure 13a, a high-density current was obtained for the graphene@WS_2_, which was observed to quickly increase with increasing RHE with respect to the applied potential. Note that the highest current density obtained for the S4 WS_2_ at high applied voltage was reached by the graphene@WS_2_ sample at very low applied voltage. Hence, the addition of graphene dramatically enhanced the generated density current, six-folds, which was beneficial to HER. To produce WS reactions, a theoretical potential value of 1.23 V versus RHE is required without considering the surface overpotential and the voltage loss due to electron transport. As can be seen, the S4 WS_2_ nanosheets showed the lowest photocurrent density, of 17 μA/cm^2^, at 1.23 V versus RHE over the entire potential range. When the graphene was added, the photocurrent density increased vigorously to 95 μA/cm^2^. The onset potentials of the S4 WS_2_ and WS_2_: 1 wt% Gr nanosheets were 0.61 and 0.52 V, respectively. The cathodic shift in the onset potentials indicated an enhanced charge transport; hence, a higher separation efficiency was obtained, even at low ranges of applied potential.

Moreover, the applied bias potential efficiency (ABPE) was evaluated using the following equation:

$$ABPE=\frac{J\left(1.23-V_{\mathrm{bias}}\right)}{P_{\mathrm{light}}}$$

where *J* is the current density, *V*_bias_ is the bias potential, and *P*_light_ is the light power.

The ABPE equation translates how much the cell device using the processed samples as photoanodes is able to produce ionization current under an external applied voltage at constant solar irradiation. Here, the light power was AM1.5G. The applied potentials were converted into the corresponding potential versus RHE using the Nernst equation. The ABPE findings given in Figure 13b indicate that the WS_2_/ITO photoanode exhibited an ABPE of 0.79% at around 0.75 V versus RHE, while the WS_2_: 1 wt% Gr/ITO photoanode reached 4.11% at the same voltage versus RHE. This increase in ABPE in the graphhene@WS_2_ sample represented a fourfold-higher performance than that of the pristine WS_2_ nanosheets. This underlines the beneficial effect of WS_2_ loaded with graphene on photocatalytic WS reactions.

**Figure 14:**
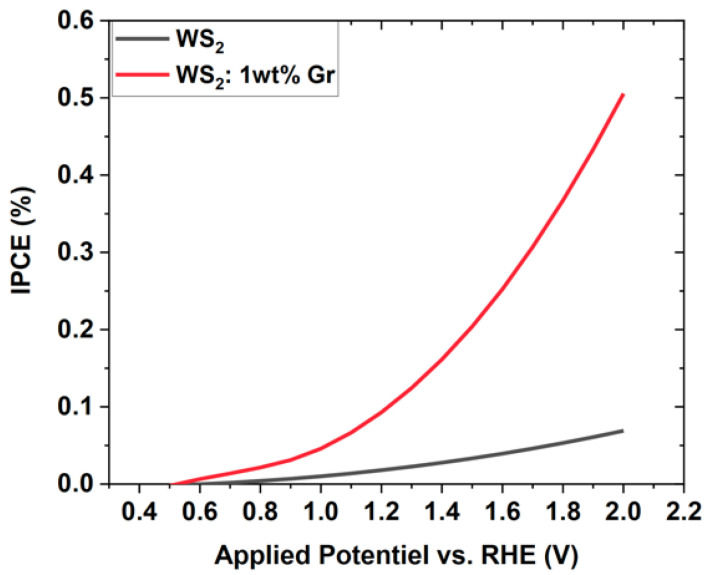
Variation in the incident photon-to-current efficiency (IPCE) as function of applied potential (V vs. RHE) calculated for neat WS_2_ NSs and WS_2_: 1 wt% Gr.

To examine the photoresponse of the photoanode WS_2_: 1 wt% Gr/ITO over time, the transient photocurrent was recorded at 0 V of bias with the light on/off cycles at AM1.5G/cm^2^, using a monitored mechanical shutter. The results depicted in Figure 13c demonstrate a fair stability profile over more than 160 s and the fast response of the excited photoanode over a duration of less than 10 s and a dwell time of 20 s. The stability of the current density was screened by the steady-state measurements of the current density generated (Figure 13d). The photocurrent stability of the WS_2_: 1 wt% Gr photoanode was examined at a bias potential of 0 V under AM1.5G illumination. A fast decay occurred for the first 100 s. Subsequently, a plateau was recorded, indicating that the photocurrent had reached a value of 0.4 nA/cm^2^ over the following 100 s. During this time, the photogenerated electrons were transferred to the Pt counter electrode to boost the HER. Consequently, the generated holes could actively participate in the oxidation process at the photoanode site. In our case, the initiation of the WS experiment induced the accumulation of the photogenerated holes at the photoanode site, since no bias potential was applied. These holes could barely be scavenged by water molecules, and recombination occurred, resulting in a decrease in the photocurrent with time. After about 100 s, the rate of generation and consumption of the holes became constant and the photocurrent is stable [15]. Furthermore, we accounted for the efficiency of our photoelectrochemical process for the neat WS_2_ NSs and WS_2_: 1 wt% Gr by evaluating the incident photon-to-current efficiency (IPCE), which gives a good estimation of the number of produced electrons with respect of the number of incident photons. The IPCE was determined by the following expression: IPCE (%) = *J* × V/incident Power. The obtained IPCE was plotted against the applied potential (V vs. RHE) and depicted in Figure 14.

Figure 14 shows an increase in IPCE for both samples as a function of the applied bias. The IPCE reached 0.1% for the neat sample, whereas it approached more than 0.5% for the WS_2_: 1 wt% Gr. Therefore, the incorporation of the graphene dramatically enhanced the IPCE, which exhibited an exponential function profile. This indicates that the graphene provided additional electrons circulating in the photochemical cell, which is further proof of the beneficial effect of graphene in enhancing photochemical reactions.

**Figure 15:**
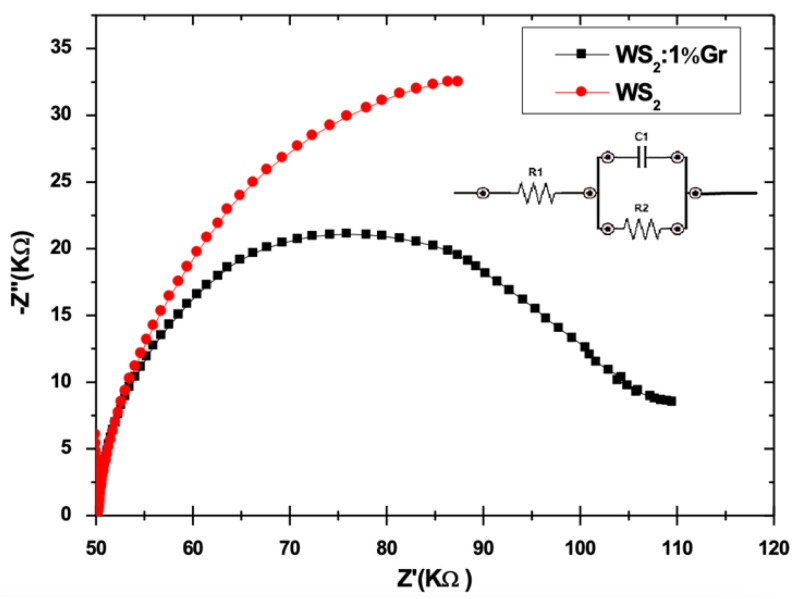
EIS measurements of WS_2_ and WS_2_: 1 wt% Gr samples. The inset is the equivalent circuit used to extract the resistance of both samples.

To further examine the performance of the WS_2_: 1 wt% Gr photoanode, an EIS experiment was carried out to evaluate its charge transfer capabilities. The EIS was conducted on a three-electrode cell and deionized water electrolyte. A Pt fishnet and Ag/AgCl were utilized as the counter and reference electrodes, respectively. The applied voltage was set to 1 V, with a frequency sweep in the range of 0.01 Hz–100 KHz under visible light irradiation at 100 mW/cm^2^ of power density. The exposed surface area of all the samples was set to 1 cm^2^. Nyquist plots of the optimized WS_2_ and WS_2_:1 wt% Gr and the corresponding equivalent electrical circuit are shown in Figure 15.

The comparison of the EIS measurements with the equivalent electrical circuit indicated the higher resistance of the S4 WS_2_ nanosheets (45.8 KΩ) compared to the very low resistance obtained for WS_2_: 1 wt% Gr (10 KΩ). This result clearly shows that the WS_2_ loaded with graphene promoted a fourfold better charge transfer compared to the neat WS_2_ sample. This performance undoubtedly suggests that graphene@WS_2_ is more suitable for use as a photoanode for HER.

In summary, in contrast to previously reported work [2,27], the materials used in the present study were produced using a one-pot fabrication process yielding a nanocomposite material made of WS_2_ NSs and graphene. Our findings demonstrate that processing composite materials based on TMD materials combined with a semimetal achieves four-to-five-times better current density at 1.23 V (V vs. RHE), which is necessary to drive WS reactions and hydrogen-evolution reactions. This contrasts with the findings of a previous study [23], in which the r-GO semiconductor was mixed with TMDs to produce heterostructures exhibiting good performances at lower applied potentials (i.e., −0.3 V for WS_2_/rGO and −0.4 V for neat WS2). These values remain low compared to the theoretical potential of 1.23 V required to achieve WS and HER.

## 4. Conclusions

Nanocomposite materials consisting of graphene and WS_2_ were produced in order to be used as highly efficient photoanodes for hydrogen production via a water-splitting reaction. The successful fabrication of the optimized WS_2_ nanosheets as well as the graphene@WS_2_ with two graphene contents was confirmed by several combined techniques, including XRD, Raman and FTIR spectroscopies, SEM, and HRTEM. Our main findings indicate that the sample WS_2_ with 1 wt% graphene exhibited a broadband visible light absorption reaching 98% and an appropriate band gap of 1.75 eV for water-splitting reactions. These outstanding optical properties led to an enhancement of the photogenerated electrons and to a higher charge transfer, as recorded by the photochemical and electron impedance spectroscopy measurements at ambient conditions. Furthermore, associating graphene with WS_2_ at only 1 wt% content led to an increase in the current density from 17 to 95 μA/cm^2^ at 1.23 V versus RHE under AM1.5G illumination with ABPE, which was fourfold higher than the pristine WS_2_ nanosheets. Hence, the graphene@WS_2_ could be considered a desirable high-efficient photoanode for hydrogen-evolution reactions.

## Figures and Tables

**Table 1:** The precursor masses used to prepare each WS_2_ sample.

Sample	S1	S2	S3	S4
Thiourea (g)	4.5	5	5	7
WO_3_ (g)	0.23	0.23	0.22	0.2

## Data Availability

All data and analyses are available upon request to the corresponding author.
